# Concurrent Validity of Physiological Cost Index in Walking over Ground and during Robotic Training in Subacute Stroke Patients

**DOI:** 10.1155/2014/384896

**Published:** 2014-05-19

**Authors:** Anna Sofia Delussu, Giovanni Morone, Marco Iosa, Maura Bragoni, Stefano Paolucci, Marco Traballesi

**Affiliations:** Santa Lucia Foundation, I.R.C.C.S., Via Ardeatina 306, 00179 Rome, Italy

## Abstract

Physiological Cost Index (PCI) has been proposed to assess gait demand. The purpose of the study was to establish whether PCI is a valid indicator in subacute stroke patients of energy cost of walking in different walking conditions, that is, over ground and on the Gait Trainer (GT) with body weight support (BWS). The study tested if correlations exist between PCI and ECW, indicating validity of the measure and, by implication, validity of PCI. Six patients (patient group (PG)) with subacute stroke and 6 healthy age- and size-matched subjects as control group (CG) performed, in a random sequence in different days, walking tests overground and on the GT with 0, 30, and 50% BWS. There was a good to excellent correlation between PCI and ECW in the observed walking conditions: in PG Pearson correlation was 0.919 (*p* < 0.001); in CG Pearson correlation was 0.852 (*p* < 0.001). In conclusion, the high significant correlations between PCI and ECW, in all the observed walking conditions, suggest that PCI is a valid outcome measure in subacute stroke patients.

## 1. Introduction

In stroke survivors cardiorespiratory reconditioning represents a challenge to improve patients' mobility and quality of life, especially for those who regain deambulation in the community [1].

The stroke survivor reduction of cardiovascular fitness is a real problem limiting patients' return in community life. This problem comes out because more than 75% of patients affected by a stroke have a cardiovascular disease [2] and because after a stroke patients reduce their mobility. As recommended by the American Heart Association, moderate aerobic training is useful in subacute stroke condition to avoid deconditioning [3], and several authors during the last 10 years documented the importance of an aerobic training in stroke survivors in terms of reducing insulin resistance, improving lipid profile and glucose tolerance, and improving cognitive function [4–6].

For these reasons electromechanical assisted and robotic machines providing body weight support (BWS) were made to train nonambulatory patients, with less demand for the physiotherapist, and should be useful for increasing the amount of walking exercise avoiding deconditioning. In fact Chang et al. demonstrated that more than two weeks of Lokomat training improved cardiovascular fitness early after stroke [7].

The Gait Trainer (GTII, Rehastim, Berlin) [8, 9] is one of these machines and its positive effect on walking ability was well documented [10] especially in more severe patients [11, 12]. During GT exercise it is important to know patients' cardiac demand and oxygen consumption to train patients in a safe manner to improve the reconditioning across the therapy session.

Oxygen consumption and energy cost of walking (ECW) have been widely used in the literature investigating the efficacy of interventions for improvement of walking capability. It has been reported that gas exchange analysis is a reliable method after stroke [13, 14]; nevertheless ECW measurement is generally impracticable in clinical settings due to unavailability of dedicated instrumentations and expert physicians. Another method used to assess gait demand is the Physiological Cost Index (PCI), proposed by MacGregor [15, 16]. The PCI is calculated as follows: (heart rate during steady state exercise minus heart rate at rest) divided by walking speed; PCI is expressed in beats/meter and indicates the increased heart rate (HR) necessary for exercise (walking). The PCI theory has been based on the fact that, for submaximal effort, a correlation exists between HR and V'O_2_. Based on this correlation, PCI has the potential to represent an easy and cheap index of ECW for a given subject, useful for clinicians that have no other more expensive and sophisticated devices as, for example, portable gas analyzer.

The correlations between PCI and V'O_2_ have been investigated in amputees (children and adults) [17, 18], children with cerebral palsy [19], adults with spinal cord injuries [20], and healthy adults [21]. Also PCI has been reported as outcome measure, in several studies, after interventions in persons with cerebral palsy [22], spinal cord injury [23, 24], rheumatoid arthritis [25], stroke [26–28], and acquired brain injury [29].

A few and conflicting data are reported in the literature about validity and reliability of PCI in stroke population: Danielsson et al. concluded that the PCI showed limited reliability and validity as a measure of energy cost after stroke, even if it would be useful as a simple measure for patients in clinical situation [30]; Fredrickson et al. reported that the PCI can be used as a proxy index for the oxygen cost of walking in subjects after stroke [31]; in a more recent work Danielsson et al. [32] estimated the ECW of subjects with motor impairment late after stroke by means of PCI.

It has to be considered that heart rate measurement could be affected by altered vagal or sympathetic regulation, secondary to brain injury [33–36] or medication. Nonetheless, it would be of clinical interest to assess the PCI method in a sample of persons with (subacute) stroke to test its suitability as a simple, inexpensive measure of energy cost.

Concerning validity, correlations between the PCI and ECW were reported by Bowen et al. [19] in a study on children with cerebral palsy, where a correlation coefficient of 0.50 was found. An extremely high correlation (*r* = 0.99) was found between HR and V'O_2_ by Rose et al. [37] in two- minute walk tests conducted at different speeds. Engsberg et al. [17] reported that the vertical displacement of the pelvis, the PCI, and HR were adequate tools in the assessment of energy expenditure. In a study on patients with spinal cord injury, Ijzerman et al. [20] concluded that the ability of the PCI to detect changes (longitudinal validity) was good (*r* = 0.86). To our knowledge there are no studies about assessment of ECW, during overground walking or during walking on the GT, by means of PCI in subacute stroke patients. Thus, in the present study the aim was to establish whether PCI is a valid indicator in subacute stroke patients, and in healthy age- and size-matched subjects, of ECW in different walking conditions, that is, over ground and on the GT with BWS. To accomplish the aim, the study tested if correlations exist between PCI and ECW, indicating validity of the measure and, by implication, validity of PCI. Finally, in order to provide information regarding energy demand during robotic training with BWS oxygen consumption data in different GT BWS walking conditions have been quantified in MET.

## 2. Methods

Patients with stroke in a rehabilitation department were asked to volunteer for the study (patients group (PG)). The inclusion criteria were first time stroke at least 6 months previously, 18 to 65 years of age, hemiparesis, stable heart condition, and walking ability without assistance for 5 minutes (or, if necessary, with a walking aid or orthosis). Exclusion criteria were severe cardiac disease or arrhythmia, pain during walking, walking impairment other than stroke-induced, and inability to understand information or follow instructions. An age- and body-size-matched healthy control group (CG) was also recruited. The study was approved by the local ethics committee. All participants were informed before they signed the consent form to take part in the study. All study participants performed an overground walking test (OGWT) and 3 walking tests on the GT with three different percentages of body weight support (BWS), namely, 0% BWS, 30% BWS, and 50% BWS (GTWT-0% BWS, GTWT-30% BWS, and GTWT-50% BWS). Each participant performed one test per day in four consecutive days in a random sequence. For the OGWT, participant had to walk forth and back along a 20 m linear course at a self-selected walking speed. Patients were allowed to use their walking aids (e.g., cane) if necessary. Also on the GT, walking speed was self-selected during the first minute of walking and then remained unchanged until the walking test end. During all tests participants wore a portable breath by breath gas analyzer K4b^2^ (Cosmed, Italy) to assess oxygen consumption (V'O_2_) and a heart rate monitor (Polar Electro Oy, Finland) to collect heart rate (HR) data. Each WT (OGWT and GTWT) lasted at least 5 minutes to allow reaching and maintaining a cardiac and metabolic steady state (SS).

As baseline data the mean values of the last 3 minutes of a 10-minute resting condition recording were considered while the SS phase data were calculated as the mean value of the data collected in the last two minutes of data recording during each walking test.

Mean walking speed during OGWT was calculated as the ratio of distance to time; thus, the walking speed obtained in the last 2 min of data collection was considered.

The PCI was calculated as follows:

(1) $$\begin{array}{c} \text{PCI}=\frac{\text{SSHR}\;\left(\text{beats}/\min\right)-\text{Resting}\;\text{HR}\;\left(\text{beats}/\min\right)}{\text{walking}\;\text{speed}\left(\text{m}/\min\right)}. \end{array}$$

### 2.1. Statistical Analysis

Mean and standard deviation were computed for all the measured parameters. We choose to use repeated measure analysis of variance because the measurements were continuous and because this analysis allows for comparing at the same time within- and between-subjects factors. A repeated measures ANOVA was carried out to assess differences within (walking conditions: over ground, GTWT-0% BWS, GTWT-30% BWS, and GTWT-50% BWS) and between (group: PG, CG) subjects factors. Walking conditions and group were considered as main factors in this analysis; thus the comparison between walking conditions was performed by including all subjects in the two groups; the group comparisons were performed by including all walking conditions. The level of significance for the ANOVA analysis was set at *p* < 0.05. When ANOVA revealed statistically significant results, post hoc comparisons were carried out with Bonferroni correction. To assess correlations between PCI and ECW a Pearson correlation was calculated.

## 3. Results

Six patients with hemiplegia due to stroke (age: 66 ± 15 y; time since stroke: 8 ± 3 weeks; four men) and 6 healthy age- and size-matched subjects as CG (age: 76 ± 7 y; six men) were enrolled in the study. PG and CG mean body mass and stature were 66 ± 6 kg and 164 ± 7 cm, 76 ± 7 kg, and 173 ± 3 cm, respectively. Only one stroke subject needed aid for OGWT; all patients were able to reach and maintain SS phase, as described in the protocol.

The mean self-selected walking speed of PG during OGWT was 1.25 ± 0.51 km/h; in the same WT CG walked at 3.60 ± 0.44 km/h, a speed significantly higher than that chosen by PG (*p* < 0.001). On the GT the mean self-selected walking speeds of PG for GTWT-0% BWS, GTWT-30% BWS, and GTWT-50% BWS were 1.53 ± 0.18, 1.50 ± 0.17, and 1.51 ± 0.17 km/h, respectively. CG mean self-selected walking speeds during GTWT-0% BWS, GTWT-30% BWS, and GTWT-50% BWS were 1.57 ± 0.16, 1.54 ± 0.12, and 1.62 ± 0.22 km/h, respectively. No differences were observed between groups in the GTWTs speeds. Within-group analysis showed that OGWT PG speed did not differ significantly from that on the GT, while for CG the OGWT speed was significantly higher than that reached at each GTWT, *p* < 0.001.

**Figure 1:**
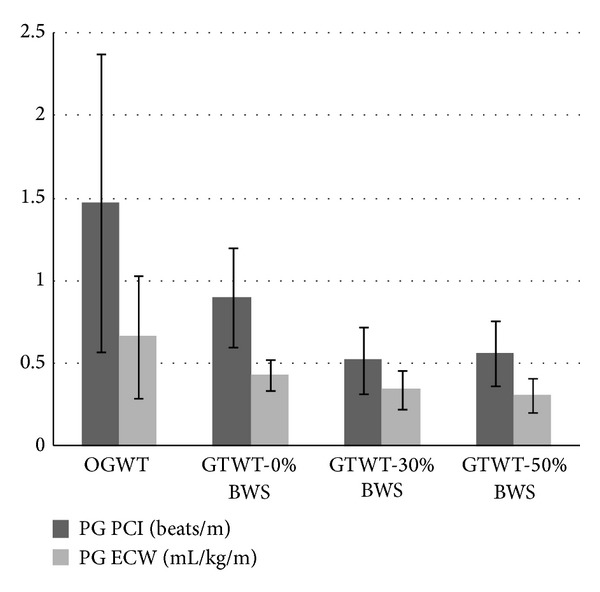
PCI and ECW of PG in the observed walking conditions.

**Figure 2:**
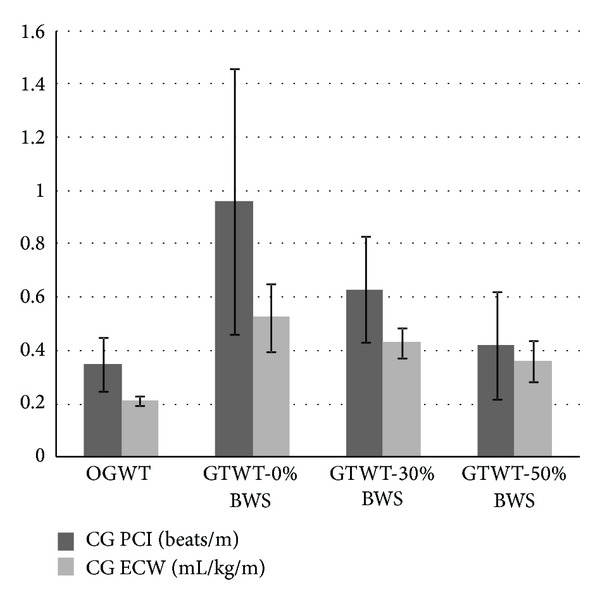
PCI and ECW of CG in the observed walking conditions.

Figures 1 and 2 show PCI and ECW data of PG and CG, respectively, in all the observed walking conditions. PG PCI mean values accounted for abnormally elevated values in OGWT compared to CG (1.45 ± 0.87 versus 0.35 ± 0.06 beats/m); the difference between groups was statistically significant (*p* = 0.012, with Bonferroni correction). Also ECW PG data of OGWT were higher than those of CG (0.66 ± 0.37 versus 0.21 ± 0.02 mL/kg/m), with statistical significance (*p* = 0.015, with Bonferroni correction). Further, it has to be noted that for PG OGWT has the significantly highest values for both PCI and ECW, while for it CG is the contrary (*p* < 0.02).

As can be seen in Figures 1 and 2, for both groups on the GT the improvement in BWS is paralleled by a decrease of PCI and ECW. On the GT, for all the observed walking conditions (i.e., with the several percentages of BWS), there were no differences between groups. At the within- group analysis PG showed statistical differences between GTWT-30 and -50% BWS versus GTWT-0% BWS, *p* < 0.02, while CG showed significantly higher values between GTWT-0, -30, and -50% BWS versus OGWT (*p* < 0.02). Further, due to the reduced sample size, we have also used nonparametric statistics (Friedman's analysis) for confirming (or not) the results of parametric one.

**Figure 3:**
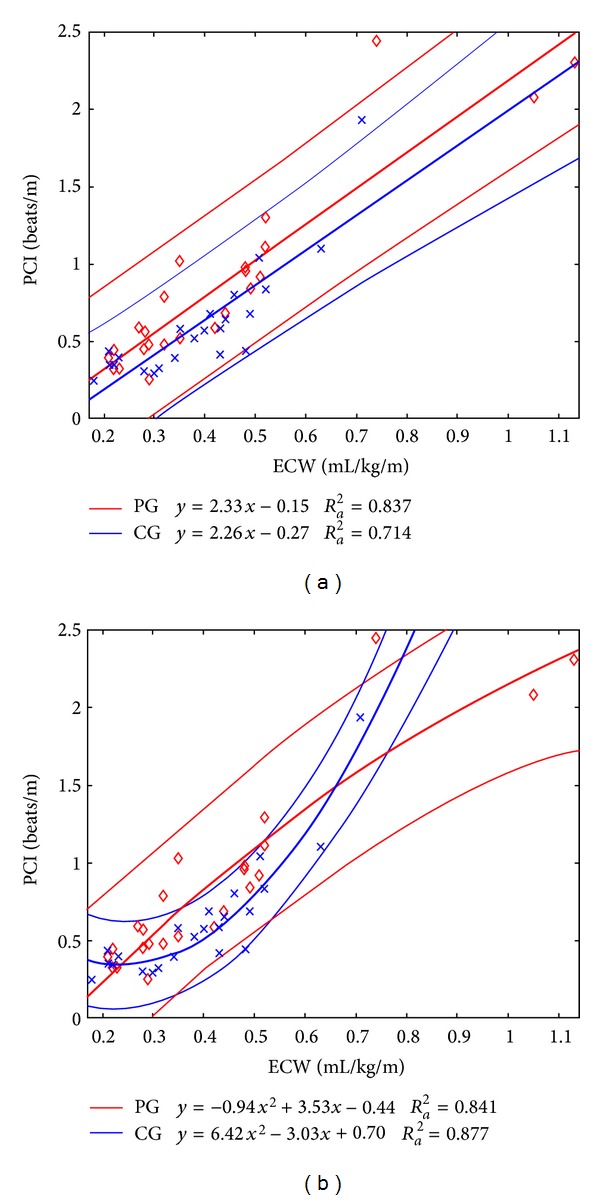
Correlations between PCI and ECW, in all the observed walking conditions (over ground, on the GT with 0, 30, and 50% BGWS), of PG (red diamonds) and CG (blue crosses). On the left (a) are first-order polynomial fitting lines and on the right (b) second-order polynomial fitting lines (with the same color of data). Thin lines represent the 95% confidence interval for all the fits. Equations of fits and values of adjusted *R* ^2^ are also reported.

As reported in Figure 3, there is a good to excellent correlation between PCI and ECW, in the observed walking conditions: in PG Pearson correlation was 0.919 (*p* < 0.001); in CG Pearson correlation was 0.852 (*p* < 0.001). Furthermore, the two fitting lines based on a first-order polynomial model resulted to be very similar in the two groups. Conversely, although quadratic regressions improved the fitting of data in terms of adjusted *R* ^2^, the curve for healthy subjects diverged from that of patients, for which the increment of the polynomial fit order slightly varied the adjusted *R* ^2^.

Table 1 reports energy expenditure data, expressed as MET, and *p* values, of both groups in all the observed conditions.

PG had the highest MET values at OGWT, while CG had the highest value at GTWT-0% BWS. On the GTWT both groups showed a decrease of energy expenditure with the increase of BWS. As can be noted the CG always showed values higher than PG; no statistical differences were observed between groups. According to work classification, for both groups, OGWT and GTWT-0% BWS accounted for a moderate intensity work, while GTWT-30% and GTWT-50% BWS resulted as light job [38].

## 4. Discussion

The lack of sophisticated and expensive instrumentations and of ad hoc trained physicians/physiotherapists in clinical settings determines the need of cheap and easy methods to obtain valid outcome measures. The widely diffused possibility to collect heart rate may allow for the implementation of outcome measure based on heart rate data. Thus the present study aimed at verifying the validity of PCI as cheap and easy outcome measure in subacute stroke patients. Further, the increasing diffusion of robotic machines providing BWS [7, 11, 39], like the GT, induced us to investigate the validity of PCI also during training on the GT.

To accomplish this aim, the PCI and the ECW in a PG and in an age- and size-matched CG were determined in several walking conditions, namely, OGWT, GTWT-0%, GTWT-30%, and GTWT-50% BWS; then the correlation between PCI and ECW data was determined.

The high significant correlation for PG (*r* = 0.9191, *p* < 0.001) and for CG (*r* = 0.852, *p* < 0.001) suggests the possibility of using PCI as valid outcome measure in subacute stroke patients. Further, related to OGWT, PCI was able to discriminate stroke patients from healthy subjects (1.45 ± 0.87 versus 0.35 ± 0.06 beats/m, *p* = 0.012), similarly to ECW (0.66 ± 0.37 versus 0.21 ± 0.02 mL/kg/m, *p* = 0.015). Our PG OGWT data are in line with those reported by Mossberg [29] for PCI, while their ECW data were lower than ours (0.374 ± 0.203 mL/kg/m) for stroke subjects. Further, also Mossberg [29] found a significant good correlation between PCI and ECW in stroke patients during walking on treadmill. PCI and ECW data reported by Stein et al. [28] are lower than our PG OGWT data, probably because in the study protocol of Stein et al. for walking test a treadmill was used provided with hand support, and the protocol allowed for a light hand support during walking. This could have reduced the energy and cardiac demand in comparison to our study protocol.

As reported in Figure 1, for PG OGWT had the highest values for both PCI and ECW; besides, on the GT the improvement in BWS was paralleled by a decrease of PCI and ECW. On the GT the same trend of PCI and ECW was observed for CG, but CG, differently from the PG, had the lowest PCI and ECW data in OGWT, as can be seen in Figure 2. This last result is due to the fact that CG had no limitations in performing OGWT, considering that CG was healthy and OGWT represented a habitual motor task, while for PG OGWT represented something to be regained, hard to perform in comparison to GTWTs. It has to be noted that CG had on the GTWTs walking speed values close to those of the PG, because GT permits a maximal walking speed of 2 Km/h [8].

The similarity of PCI and ECW trends on the GTWTs, with the different BWSs, between PG and CG is more evident in Figure 3, where the correlation between PCI and ECW is reported for both groups. The PCI has a high correlation with ECW that indicates its validity either on the OGWT or on the GT with different BWS. As ECW, also PCI is able to detect differences between PG and CG (1.45 ± 0.87 versus 0.35 ± 0.06 beats/m, *p* = 0.012). On the GT for all walking conditions (i.e., with 0, 30, and 50% BWS) neither ECW nor PCI revealed differences between groups. This fact could be due to the light intensity [36] of the job performed, particularly at GTWT-30% and GTWT-50% BWS. Nevertheless both ECW and PCI, at the within-group analysis, revealed statistical significant differences among GTWT BWS conditions: PG showed statistical differences between GTWT-30 and 50% BWS versus GTWT-0% BWS, *p* < 0.02, while CG showed significantly higher values between GTWT-0, -30, and -50% BWS versus OGWT (*p* < 0.02).

As a further result, as reported in Table 1, PG and CG had the highest MET values at OGWT and GTWT-0% BWS, respectively, and on the GTWTs both groups showed a decrease of energy expenditure with the increase of BWS. Besides, for both groups, OGWT and GTWT-0% accounted for a moderate intensity work, while GTWT-30% and GTWT-50% resulted as light job. This last result, in accordance with PCI and ECW data, further confirms that training on the GT with 30–50% of BWS is less energy demanding and suggests that it could be a safer walking rehabilitation tool with respect to the traditional one conducted over ground.

### 4.1. Study Limitation

The main limitation of the study was small sample size. This also limited the possibility to take into account possible confounding factors such as age or basic gait speed. It has to be considered that it is hard to convince patients who have yet a lot of problems and discomfort to be engaged in a study like ours. Not so many people are prone to be engaged in several measures that are not invasive but fastidious and that need patients' active participation. However, our data adds information to previous findings, useful in clinical settings.

## 5. Conclusion

The high significant correlations between PCI and ECW, in all the observed walking conditions, suggest that PCI is a valid outcome measure in subacute stroke patients. Also, PCI is comparable to ECW in its ability to discriminate between stroke patients and healthy subjects in overground walking test.

## Figures and Tables

**Table 1:** MET of PG and CG in the observed walking conditions.

	REST	OGWT	GTWT-0% BWS	GTWT-30% BWS	GTWT-50% BWS
PG	0.80 ± 0.27	3.3 ± 0.8	3.1 ± 0.8	2.4^∗§^ ± 0.9	2.2^∗§^ ± 0.9
CG	1.05 ± 0.21	3.6 ± 0.6	3.9 ± 1.2	3.1^∗§^ ± 0.5	2.7^∗§^ ± 0.6

Data are reported as mean and standard deviation. Significant difference of post hoc analysis: *with respect to overground and ^§^with respect to GTWT-0% BWS.
